# A Facile and Effective Method to Fabricate Superhydrophobic/Superoeophilic Surface for the Separation of Both Water/Oil Mixtures and Water-in-Oil Emulsions

**DOI:** 10.3390/polym9110563

**Published:** 2017-10-30

**Authors:** Feiran Li, Ziran Wang, Yunlu Pan, Xuezeng Zhao

**Affiliations:** Key laboratory of Micro-Systems and Micro-Structures Manufacturing, Ministry of Education and School of Mechatronics Engineering, Harbin Institute of Technology, Harbin 150001, China; 15B908006@hit.edu.cn (F.L.); 16B908024@hit.edu.cn (Z.W.); zhaoxz@hit.edu.cn (X.Z.)

**Keywords:** superhydrophobic, superoleophilic, oil/water separation, stabilized water-in-oil emulsion

## Abstract

Superhydrophobic/superoleophilic surfaces (water contact angle greater than 150° with low hysteresis, with an oil contact angle smaller than 5°) have a wide-range of applications in oil/water separation. However, most of the essential methods to fabricate this kind of surface are complex, inflexible, and costly. Moreover, most methods focus on separating immiscible oil and water mixtures but lack the ability to demulsify surfactant-stabilized emulsions, which is widely present in industry and daily life. In this study, a facile and effective method was developed to fabricate superhydrophobic/superoleophilic surfaces that can be easily applied on almost all kinds of solid substrates. The treated porous substrates (e.g., steel mesh; cotton) can separate oil/water mixtures or absorb oil from a mixture. Furthermore, the compressed treated cotton is capable of demulsifying stabilized water-in-oil emulsions with high efficiency. The simple, low-cost, and material-unrestricted method provides an efficient way to separate oil/water mixtures of various kinds and has great potential in energy conservation and environmental protection.

## 1. Introduction

Superlyophobic surfaces (on which a liquid’s contact angle is greater than 150° and its slide angle is smaller than 10°) and superlyophilic surfaces (on which a liquid’s contact angle is smaller than 5°) have widespread applications in energy conservation, environmental protection, and biomedical materials [1,2,3,4,5]. Amongst them, superhydrophobic/superoleophilic surfaces (water contact angle greater than 150°, with an oil contact angle smaller than 5°) have a wide range of potential applications in oil/water separation thanks to its water-repellent/oil-absorbing properties [6,7,8,9,10,11,12,13,14]. A low-energy chemical composition and rough geometrical structure are two essential requirements for fabricating superlyophobic surfaces. To fabricate superlyophilic surfaces, on the other hand, higher surface energy and roughness is desired [15,16,17]. In order to fabricate surfaces of superwettablity, effective processing methods like photoetching [18,19,20,21,22], chemical vapor deposition (CVD) [23,24,25,26], and plasma treatment [27,28,29,30,31,32,33] are widely used. These methods, however, are generally costly, material-restrictive, and unsuited for large-scale production [34,35,36,37]. Moreover, sometimes the water/oil mixture is not just immiscible; it may form a more complete mixing state of the disperse phase, called emulsion. The existence of emulsified oil and water mixtures, especially water-in-oil emulsions, has always been a severe problem in the industry and environment. Most previous studies of superhydrophobic/superoleophilic materials have good performance in separating immiscible oil/water mixtures, but have failed in separating water-in-oil emulsions in which the dispersed phase is smaller than 20 μm [38,39,40,41,42]. The separation of emulsions is much more difficult, and the separation is different from that of immiscible water/oil mixtures. Some methods have proven to be effective in separating both immiscible water/oil mixtures and emulsions by changing substrates or applying external stimulus, but the fabricating and operating process is complicated [43,44]. Therefore, methods with lower cost, more flexibility, and better applicability and efficiency for both immiscible water/oil mixtures and emulsions is highly desired for use in future widespread industrialization.

Herein, a facile and effective method was proposed to fabricate superhydrophobic/ superoleophilic surfaces simply by spraying or painting a mixed suspension on the substrate. The preparation process is feasible and can be flexibly applied on almost any solid substrate. The treated surfaces exhibit excellent superhydrophobicity and superoleophilicity. Various substrates can be used for separating oil and water mixtures in different ways. For example, treated steel mesh can separate immiscible oil/water mixtures effectively; and treated cotton can absorb oil from oil/water mixtures without loss of water. Furthermore, compressed treated cotton is capable of separating stabilized water-in-oil emulsions with high efficiency. The proposed method, which provides a shortcut to the fabrication of superhydrophobic/superoleophilic surfaces, has extensive application prospects in separating various kinds of oil/water mixtures and is an ideal solution for the growing problem of resource recycling and environmental pollution.

## 2. Experimental Section

### 2.1. Materials

Aluminum oxide (Al_2_O_3_) nanoparticles in three different diameters (30 nm, ~200 nm, 1 μm), *n*-dodecane (AR, 96%), *n*-octane (≥98%, GC), *n*-dodecane (≥99.7%, GC), 1,2-dichloroethane (≥99.8%, GC), diiodomethane (98%), and hexadecane (≥99.5%, GC) were obtained from Shanghai Aladdin Bio-Chem Technology Corporation (Shanghai, China). 1*H*,1*H*,2*H*,2*H*-Perfluorooctyltriethoxysilane and Span 80 were purchased from Shanghai Macklin Biochemical Corporation (Shanghai, China). Olive oil was purchased from Wal-Mart Stores, Inc. Diesel oil was obtained from Sinopec gas station (Harbin, China). All chemicals were analytical grade reagents and were used as received.

### 2.2. Fabrication of Superhydrophobic/Superoleophilic Surfaces

First, 1 g 1*H*,1*H*,2*H*,2*H*-Perfluorooctyltriethoxysilane was mixed with 100 g absolute ethanol solution and magnetically stirred for 20 min at room temperature until fluoroalkyl silane was hydrolyzed, and then 1 g Al_2_O_3_ nanoparticles (diameter of 1 μm), 5 g Al_2_O_3_ nanoparticles (diameter of 200 nm) and 2.5 g Al_2_O_3_ nanoparticles (diameter of 30 nm) were added to the solution with stirring for 1 h to form a homogeneous suspension. The suspension could be sprayed (using a spray gun, 20 psi) or painted on the substrates. The treated surfaces showed superhydrophobicity and superoleophilicity as soon as the surfaces were dried within several minutes.

### 2.3. Preparation of Water-in-Oil Emulsions

Stabilized water-in-oil emulsions was prepared by mixing deionized water (95 mL), oil (5 mL), and Span 80 (0.4 mL) in a beaker. Then the mixture was vibrated with an ultrasonic homogenizer (Xinzhi JY92-11N, 20 kHz frequency at 20% amplitude, Xinzhi, Ningbo, China) for 3 h, and the obtained emulsion was stable for more than 20 h in ambient environment. In this study, three kinds of emulsions were prepared with olive oil, diesel, and hexadecane.

### 2.4. Setup of Water-in-Oil Emulsion Separation

To separate water-in-oil emulsion, cotton was initially soaked in the suspension and ultrasonic vibrated for 30 min, then dried at 80 °C for 3 h until the ethanol solution had completely evaporated. The treated cotton was compressed to a density larger than 0.28 g/cm^−3^ with a medical injector (Hongda M.D., Nanchang, China). The pressure exerted on the piston was measured by a pull gage (NK300, GTYG, Co., Ltd., Shanghai, China).

### 2.5. Characterization

Scanning Electron Microscopy (SEM) (ZEISS MERLIN Compact SEM, operated at a 20 kV acceleration voltage, Carl Zeiss Jena, Germany) was employed for product characterization. Contact angles were measured at ambient temperature using an optical contact angle meter (DropMeter^TM^ Element A-60, water droplet of 6 μL, Maist, Ningbo, China), each measurement was taken at least three times. The optical images of the emulsion and the filtrate were taken using a Leica DVM6s 3D Microscope (Leica, Germany). The density of the liquid was tested by Westphal balance (YuePing PZ-D-5, Zhengzhou, China), and the measurement was performed at 20 °C. The distribution of water droplets in the emulsion was measured at room temperature by a NanoBrook ZetaPALS Potential Analyzer (Brookhaven, NY, USA).

## 3. Results and Discussion

Since the surface tension of oil is smaller than that of water, it is possible to fabricate water-repellent and oil-attractive surfaces based on the surface tension theory [45,46]. The prepared suspension contains fluorate polymer (1*H*,1*H*,2*H*,2*H*-Perfluorooctyltriethoxysilane) and Al_2_O_3_ nanoparticles in different sizes. The fluorate polymer, which contains amounts of fluoric group such as –CF_2_ and –CF_3_, can lead to a lower surface energy, while different sizes of nanoparticles form a multi-dimensional structure, which is an essential requirement of constructing superlyophobic and superlyophilic surfaces.

### 3.1. The Characterization of the Superhydrophobic/Superoleophilic Surface

**Figure 1:**
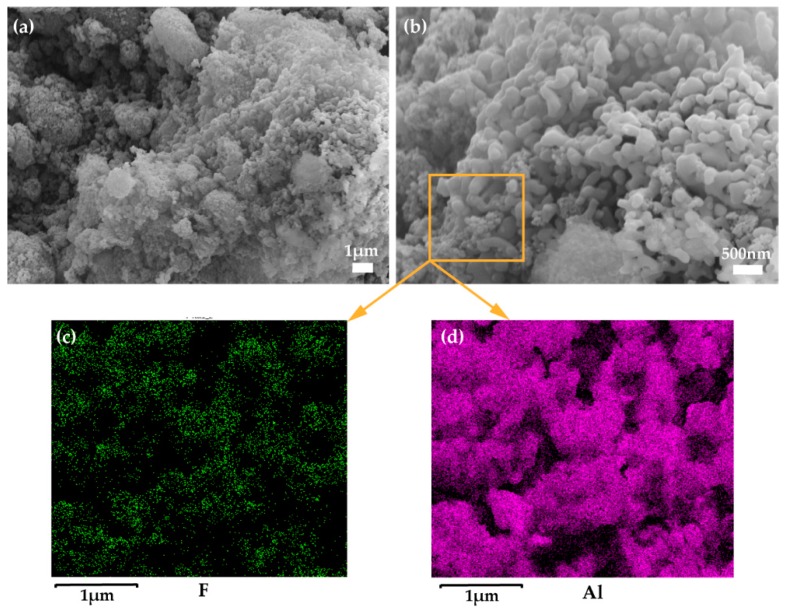
(**a**,**b**) Scanning Electron Microscope (SEM) images of a treated surface in different scales and Energy Dispersive Spectroscopy (EDS) distribution maps of (**c**) fluorine and (**d**) aluminum.

**Figure 2:**
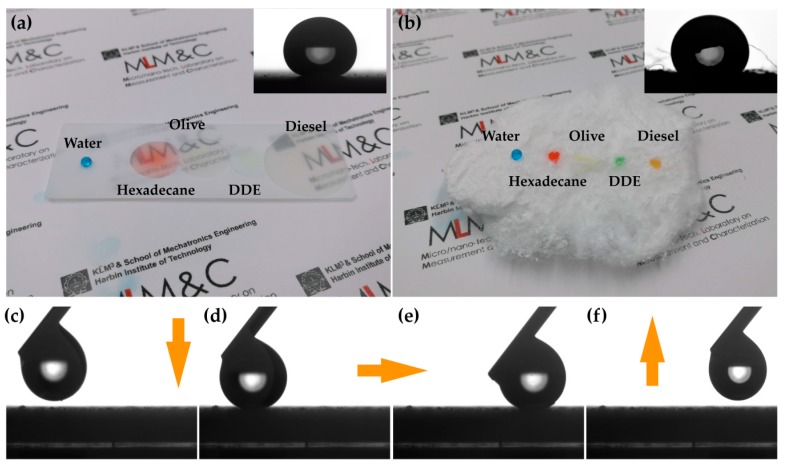
The wettability of water (dyed blue), hexadecane (dyed red), olive oil (yellow), diiodomethane (DDE, dyed green), and diesel (claybank) on (**a**) spray-treated glass and (**b**) paint-treated cotton. Insets show the shapes of the water droplets from a parallel point of view. (**c**–**f**) Continuous scene of the sliding test on the treated glass; the water droplet was pushed on the surface and slid from left to right before detachment.

The structure of the treated surface was investigated with scanning electron microscope (SEM), as shown in Figure 1a,b. It is obvious that a multi-dimensional structure was formed on the treated surface, indicating a high porosity ratio, which is indispensable for superwetting a surface based on the Cassie-Baxter theory [47]. The functionalization of the surface was also demonstrated by Energy Dispersive Spectroscopy (EDS), as shown in Figure 1c,d, and the elements of fluorine—which represents fluoric groups of –CF_2_ and –CF_3_—and aluminum—which represents the Al_2_O_3_ nanoparticles—were widely distributed on the surface, indicating the low surface energy and the high roughness [48]. The vast majority of oils have much smaller contact angles than water on flat 1*H*,1*H*,2*H*,2*H*-Perfluorooctyltriethoxysilane-treated surfaces due to their lower surface tensions, and the corresponding tendency of wettability can be expanded by constructing appropriate surface structures [49,50]. In this study, the superhydrophobicity and superoleophilicity was obtained on the rough surface created by the fluorinated nanoparticles. This method has high flexibility in fabricating superhydrophobic/superoleophilic surfaces on substrates with different hardnesses. Figure 2a,b shows the wettability of water and various kinds of oils on spray-treated glass and paint-treated cotton, while the insets show the shape of the droplets on the corresponding surfaces. The water droplets on both of the treated surfaces are nearly spherical without any contamination, while the droplets of hexadecane (27.05 mN·m^−1^, dyed red), olive oil (33.2 mN·m^−1^, yellow), diiodomethane (DDE, 50.8 mN·m^−1^, dyed green), and diesel (25.05 mN·m^−1^, claybank) wetted or permeated the surfaces. Water contact angle on the flat treated surface is ~164° and the sliding angle is smaller than 2°, which indicates a good performance of superhydrophobicity. The low hysteresis of the water droplet on the treated glass is further demonstrated in Figure 2c–f, as the water droplet slides freely on the treated surface without apparent stickiness, which further confirms the water-repellent behavior of the surface.

### 3.2. Separation of Oil/Water Mixtures

**Figure 3:**
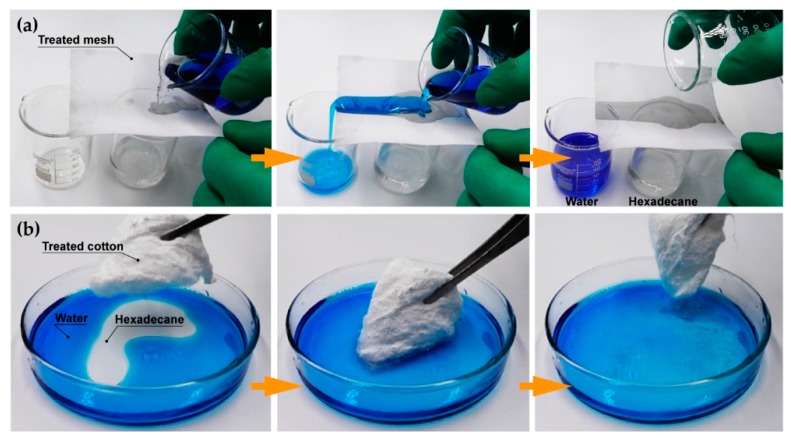
Demonstration of separating a hexadecane/water mixture with (**a**) spray-treated mesh and (**b**) soak-treated cotton. Water was dyed blue for ease of observation.

The high flexibility of the method in this study makes it possible to separate mixtures in different ways by choosing different substrates. To illustrate this idea, treated stainless-steel mesh (mesh number, 300) and degreasing cotton were used to separate immiscible oil/water mixtures, as shown in Figure 3. The mesh, spray-treated with approximate 5 mL prepared suspension on one side, was subject to the ability of water-repellency and oil-penetrability after the evaporation of ethanol solution. Figure 3a demonstrates the process of separating an immiscible hexadecane/water mixture; the hexadecane spread and permeated the treated mesh instantly, while the water (dyed blue) was resisted on the surface and slid off the mesh easily without any contamination. To test the function of absorbing oil from a mixture, a piece of degreasing cotton was initially soaked in the prepared suspension for at least 10 min, then dried at 80 °C until the ethanol solution was completely evaporated. The treated cotton could easily remove hexadecane from the mixture, as shown in Figure 3b, and there was no water (dyed blue) sticking during the whole process. The treated substrates have the same effect in separating other kinds of oil/water mixtures (e.g., *n*-octane/water, *n*-dodecane/water, olive oil/water, diesel/water, dimethicone/water). The high flexibility of the method provides an effective way to fabricate superhrodrophobic/superoleophilic materials of various forms, and thus has great potential in water pollution control and efficient oil removal.

### 3.3. Demulsification of Surfactant-Stabilized Water-in-Oil Emulsions

**Figure 4:**
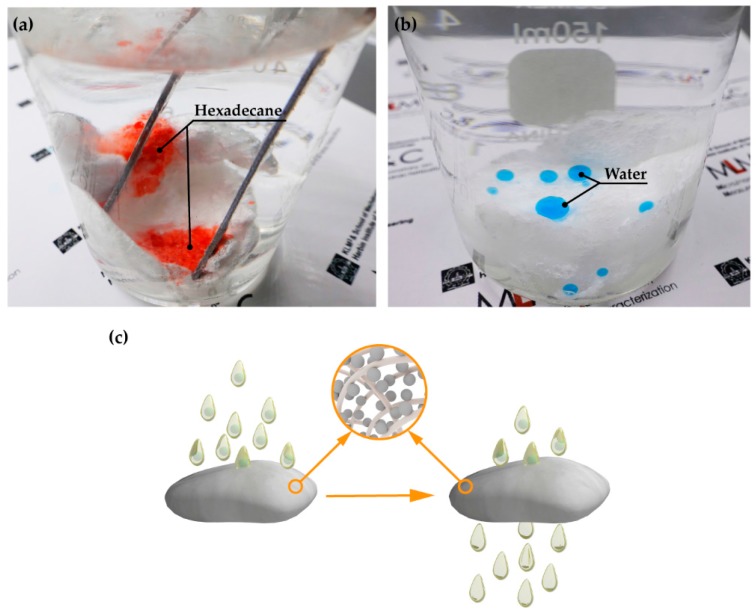
(**a**) Hexadecane (dyed red) spread and permeated the cotton in a water environment. (**b**) The superhydrophobic behavior of the treated cotton in an oil (*n*-dodecane) environment. (**c**) Schematic illustration of the demulsification mechanism of a water-in-oil emulsion.

**Figure 5:**
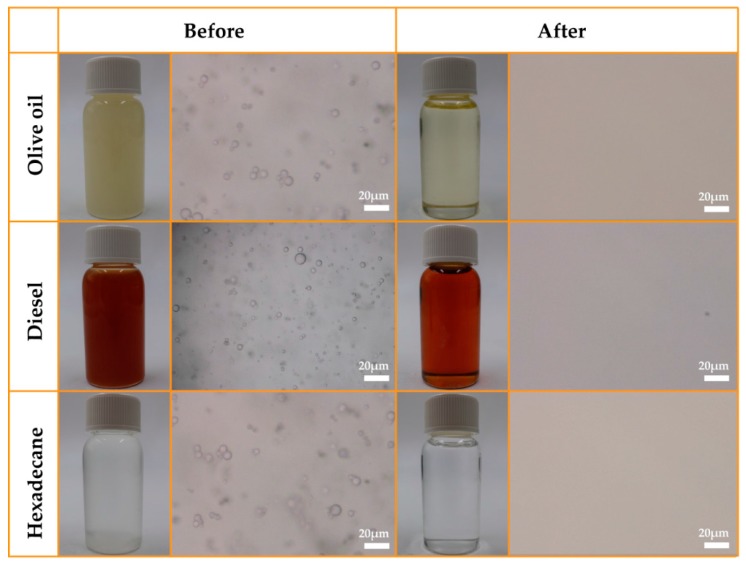
Photographs of stabilized water-in-olive oil emulsion, stabilized water-in-diesel emulsion, and stabilized water-in-hexadecane emulsion before and after separation using the treated cotton.

**Figure 6:**
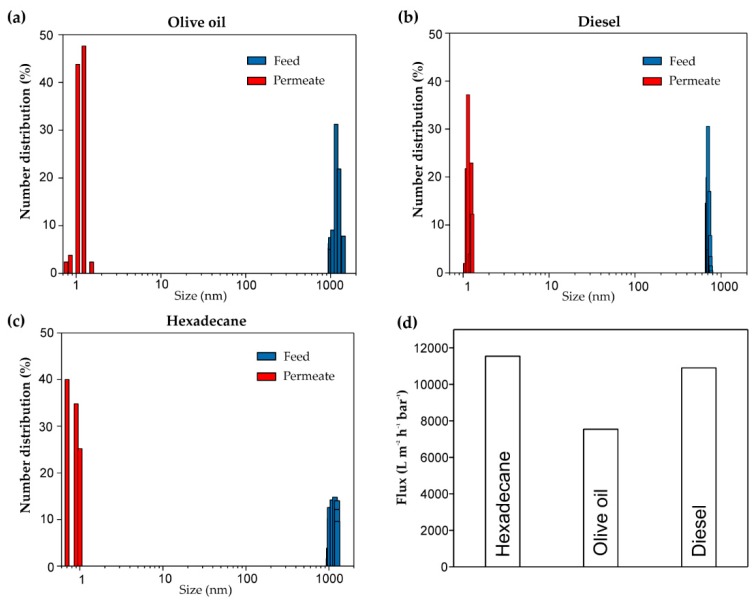
The water droplet size distribution of (**a**) stabilized water-in-olive oil emulsion; (**b**) stabilized water-in-diesel emulsion; and (**c**) stabilized water-in-hexadecane emulsion before and after separation. (**d**) Summary of the separating fluxes of three kinds of emulsions.

Emulsions, especially those stabilized with surfactants, are difficult to separate due to their micro-scale dimensions (dispersed phase <20 μm). To separate an emulsion by filtration, the filtering porous material should have the opposite wetting behavior of oil and water in a liquid environment, and the pore size of the material should also be smaller than the disperse phase [51,52,53,54]. Thanks to the flexibility of the proposed method, treated cotton can be used as a substrate to separate a stabilized water-in-oil emulsion due to its high porosity and compressibility. To illustrate this idea, the wettability of soak-treated cotton in a liquid environment was firstly tested. Figure 4a shows that oil (hexadecane, dyed red) can still spread and permeate the cotton in a water environment, while Figure 4b shows that the cotton preserves its superhydrophobicity when immersed in oil (*n*-dodecane). Moreover, the small pore size was made by compressing the cotton to a density larger than 0.25 g/cm^−3^ in an injector. The water-in-oil emulsion can be demulsified in the injector by exerting a certain force on the piston. The demulsification mechanism of the treated compressed cotton is demonstrated in Figure 4c, as the oil in the emulsion can pass through the internal gaps of the treated cotton fibers easily, while the dispersed water droplets are repelled due to the under-oil superhydrophobicity of the treated surface. Various stabilized water-in-oil emulsions can be effectively demulsified using this method. To demonstrate this effect, the quality of the feed and the filtrate of three kinds of stabilized water-in-oil emulsions (water-in-olive oil, water-in-diesel, and water-in-hexadecane) was initially tested. The results are shown in Figure 5; it is obvious that the filtrate is transparent and clear compared with the feed, which is murky and fuzzy, illustrating the good effect of the demulsification. The distribution of water droplets was further investigated by a Laser Particle Size Analyzer (ZetaPALS, NanoBrook, Brookhaven, NY, USA), as shown in Figure 6a–c, and particle size analysis demonstrated that the distribution of water droplets in the feed ranged from hundreds of nanometers to 1 μm, while the size of water droplets in the corresponding filtrate were no larger than 1.3 nm, further demonstrating the considerable effect of the demulsification. To demonstrate the efficiency of the demulsification, separation fluxes were tested, as summarized in Figure 6d. The considerable fluxes, which increased up to 11,540 Lm^−2^·h^−2^·bar^−2^, were higher than that achieved by most of the demulsifying methods using membranes [42,55,56,57,58]. Moreover, the densities of the feed and filter of different stabilized water-in-oil emulsions were also measured (Table 1), and it was found that the measured densities of the filter were highly consistent with that of the original pure oil, further demonstrating the high efficiency of the method. The treated compressed cotton (0.25 g/cm^−3^, diameter of 1.5 cm, thickness of 1 cm) kept its efficiency after the continuous separation of 3-L emulsions, indicating a strong practicability.

## 4. Conclusions

The proposed method for fabricating superhydrophobic/ superoleophilic surfaces is facile and effective; the prepared suspension containing fluoride and Al_2_O_3_ nanoparticles of different sizes can be easily coated on almost any solid substrate by spraying, painting, or soaking. Thanks to the flexibility of the method, different kinds of porous substrates such as mesh, cotton, and sponge can be easily coated and have good performance in separating immiscible oil/water mixtures in different ways. Moreover, the compressed treated cotton is capable of separating stabilized water-in-oil emulsions, which is widely present in practice, and the resultant demulsification is stable and efficient. The excellent flexibility and simplicity of the proposed method provides well-optimized solutions for environment protection and eco-saving.

## Figures and Tables

**Table 1:** Densities of feed, filter, and pure oil.

Water-in-Oil Emulsion	Density of Feed (g/cm^3^)	Density of Filtrate (g/cm^3^)	Density of Pure Oil (g/cm^3^)
Water-in-*n*-octane	0.982	0.700	0.703
Water-in-*n*-dodecane	0.984	0.753	0.750
Water-in-hexadecane	0.985	0.772	0.770
Water-in-diesel	0.990	0.840	0.840
Water-in-olive oil	0.996	0.925	0.920
Water-in-dichloroethane	1.012	1.263	1.260
